# Screening of Protease-Producing Microorganisms and Optimization of Fermentation Processes for the Efficient Preparation of *Broussonetia papyrifera* Feed

**DOI:** 10.3390/microorganisms13122722

**Published:** 2025-11-28

**Authors:** Zixing Dong, Xuehui Li, Kun Zhang, Cunduo Tang, Dandan Li, Yunchao Kan, Lunguang Yao

**Affiliations:** 1 Henan Province Engineering Research Center of Insect Bioreactor, College of Life Sciences, Nanyang Normal University, Nanyang 473061, Chinatcd530@126.com (C.T.);; 2 Henan Key Laboratory of Insect Biology, China-UK International Joint Laboratory for Insect Biology of Henan Province, Nanyang Normal University, Nanyang 473061, China

**Keywords:** protease-producing microorganisms, screening and identification, fermented *B. papyrifera* feed, efficient utilization of crude proteins, orthogonal design

## Abstract

With the rapid development of China’s livestock industry, the demand for protein feed has concomitantly increased, underscoring the importance of developing and utilizing new types of feed. As a novel and unconventional protein source, fermented *Broussonetia papyrifera* feed has become a promising alternative to traditional feeds due to its high nutritional value, abundance of bioactive compounds, and wide range of applications. However, the efficient utilization of crude proteins in *B. papyrifera* has been hindered by the currently used microorganisms and fermentation processes. In the present study, nine proteolytic bacterial strains and eight yeast and fungal strains were isolated from the root zone soil of *B. papyrifera* and screened using qualitative and quantitative methods. The protease activities of strains *Paenibacillus* sp. AY and *Rhodotorula mucilaginosa* FG were determined to be 21.95 and 55.16 U/mL, respectively. Compared with the control group, *Paenibacillus* sp. AY significantly increased the acid-soluble protein and ammonia nitrogen contents in the fermented feed by 26.7% and 12.2% (*p* < 0.05), respectively, while *R. mucilaginosa* FG enhanced them by 43.3% and 24.5%; these isolates’ effects were comparable to those of the type strains. Finally, to increase the quality of fermented *B. papyrifera* feed, the cultivation conditions were further optimized using single-factor experiments and an orthogonal design. Under optimal conditions, the acid-soluble protein content reached 7.63%, which was 27.2% higher than that of the control. Our results provide a basis for developing a novel process to efficiently utilize the crude proteins in *B. papyrifera* feed and can accelerate the application of this feed in animal production.

## 1. Introduction

Over the past two decades, China has undergone remarkable social and economic development, and it is now the world’s second-largest economy. Rapid economic growth and urbanization have led to a shift in residents’ dietary structures and nutritional preferences, leading to a large increase in demand for protein-based foods and dairy products [1,2]. To meet the demand for greater meat production, more grains are required for livestock feed [3]. The resulting competition between grain uses—human food versus livestock feed—has emerged as a new concern for China’s food security [4]. It is predicted that the supply demand gap of feed grains will reach 109.20 million tons and 179.40 million tons in 2030 [5]. To address the feed shortage, various non-conventional feed resources have been explored, such as forage sources, crop residues, agro-industrial by-products, etc. [6,7].

Paper mulberry (*Broussonetia papyrifera* (L.)) is an ecologically, economically, and medicinally important plant in the Asia-Pacific region, with features such as ease of breeding, fast growth, and strong tillering ability [8]. It has long been used in papermaking, barkcloth making, medicine, and livestock breeding. As a non-food and unconventional protein feed source approved by the Chinese Ministry of Agriculture, *B. papyrifera* has many advantages: (1) It contains abundant crude proteins, crude fat, crude fiber, polysaccharides, amino acids, vitamins, microelements, and essential trace elements, thus offering high nutritional value [8,9,10]. In particular, the crude protein content of its stem and leaves is as high as 18–22%, which is similar to that of alfalfa (~20%) [8]. (2) It produces numerous bioactive compounds, such as flavonoids, flavanes, polyphenols, alkaloids, and broussopapyrin A, which confer protective effects to livestock and poultry, including antiviral, antimicrobial, anti-inflammatory, anticancer, antioxidant, antinociceptive, and anti-tyrosinase activities [11,12,13]. (3) Using paper mulberry as an alternative to traditional protein feed can not only improve the digestibility of feed but also modulate the growth performance, gut microbiota, and immunity of monogastric animals and ruminants [14,15,16]. Being a feedstuff with broad environmental adaptability, abundant nutrients, high bioactive compound content, and a wide application range, paper mulberry has become a promising substitute for traditional protein feeds [17].

Although the crude protein content of *B. papyrifera* is high, its amino acid composition is not balanced, its palatability is low, and it is poorly digested by animals when fed to them directly [18]. Therefore, *B. papyrifera* feed is prepared using microbial fermentation technology, which increases the acid-soluble protein fraction that can be easily absorbed by animals. The microorganisms and corresponding cultivation conditions play key roles in this process. Various commercially available type strains and fermentation processes have been adopted to increase the digestibility of crude proteins [19], but to date, their utilization efficiency in *B. papyrifera* feed remains low. Hence, it is necessary to identify appropriate microbial strains and establish an optimal fermentation process for the preparation of *B. papyrifera* feed.

In the present study, the root zone soil of *B. papyrifera* collected from our university was used as the starting material, and qualitative and quantitative techniques were adopted to screen microbial strains with high protease activity. Then, the effects of the obtained strains on the acid-soluble protein content were investigated to choose appropriate candidates. After identifying the strains through 16S rRNA sequencing, their cultivation conditions were optimized using single-factor experiments and an orthogonal design to increase the utilization efficiency of crude proteins in the fermented *B. papyrifera* feed. Our results not only establish an optimal process for the efficient preparation of *B. papyrifera* feed but also contribute to alleviating the protein feed supply crisis.

## 2. Materials and Methods

### 2.1. Chemical Reagents, Microbial Strains, and Culture Media

The Folin–Ciocalteu phenol reagent and casein were bought from Sangon Biotech Co., Ltd. (Shanghai, China). Rose Bengal medium, nutrient broth medium, malt extract broth, and skim milk powder were products of Solarbio Life Sciences (Beijing, China). Paper mulberry (*B. papyrifera*) leaf powder was supplied by Sheqi Jingou Agriculture and Animal Husbandry Co., Ltd. (Nanyang, China). Bacterial, yeast, and fungal genomic DNA extraction kits were purchased from TransGen Biotech Co., Ltd. (Beijing, China). All other chemicals used in this study were commercially available in analytical grade.

*Bacillus subtilis* AS 1.398 and *Saccharomyces cerevisiae* S288C were stocked in our laboratory, while *Aspergillus oryzae* RIB40 was kindly provided by Associate Professor Bin He from Jiangxi Science and Technology Normal University. Enrichment medium consisted of 20 g of paper mulberry leaf powder and 1 L of distilled water. Medium containing 15 g of skim milk powder, 15 g of agar, and 1 L of distilled water was used to screen protease-producing bacteria, while Rose Bengal medium was employed to isolate yeasts and filamentous fungi. *Paenibacillus* sp. strains were grown in TYC medium consisting of 15 g of tryptone, 5 g of yeast extract, 50 g of sucrose, 20 g of sodium acetate, 1 g of NaCl, 2 g of NaHCO_3_, 0.1 g of Na_2_SO_4_, 0.2 g of L-cysteine, 2 g of Na_2_HPO_4_·12H_2_O, and 1 L of distilled water, pH 7.3. All media were autoclaved at 121 °C for 15 min.

### 2.2. Primary and Secondary Screening of Protease-Producing Microorganisms

Fresh samples were collected from the root zone soil of *B. papyrifera* in the vicinity of the College of Physical Education, Nanyang Normal University, Nanyang, Henan Province, P. R. China (32.993422° N and 112.536167° E). To isolate protease-active bacteria, all the samples were weighed (5 g), added to 500 mL conical flasks containing 100 mL of sterile saline, and shaken vigorously for 30 min. Afterward, 5-milliliter samples were taken, inoculated into enrichment medium, and incubated at 37 °C for 24 h. The traditional 10-fold dilution technique was adopted to isolate protease-harboring microorganisms using the agar plate method with 10^−1^ to 10^−6^ dilution factors. From each of the test tubes labeled 10^−4^, 10^−5^, and 10^−6^, 0.2 mL was evenly spread onto plates supplemented with the screening medium and subsequently cultivated in an incubator. During the incubation period, the morphological characteristics of the growing microorganisms were monitored. In addition, the ratio of clearance zone diameter (D) to colony diameter (d) was determined [20], and strains with a large ratio were selected and then isolated at 37 °C to obtain pure colonies using the streak plate method. For the primary screening of yeasts and fungi, all the samples were also cultured in the enrichment medium. Thereafter, they were inoculated into Rose Bengal medium and incubated at 30 °C for 3 days.

Selected bacterial isolates were grown in 3 mL of nutrient broth medium and incubated at 37 °C and 200 r/min for 24 h. Subsequently, 1% (*v*/*v*) inocula of the growth cultures were again inoculated into 20 mL freshly prepared nutrient broth medium and subjected to 24 h of shaking cultivation under the same conditions. For yeast and fungal strains, malt extract broth medium was used, and the strains were incubated at 30 °C and 200 r/min for 3 d. After incubation, all cultures were centrifuged at 12,000 rpm and 4 °C for 10 min, and the supernatants were collected and assayed using Chinese national standard GB/T 23527-2009 [21] to determine their protease activities. Briefly, 0.5 mL of enzyme extract was combined with 0.5 mL of casein solution (10 g/L) and incubated at 40 °C for 10 min. The reaction was then stopped by adding 1 mL of 0.4 mol/L trichloroacetic acid, followed by 1 min centrifugation at 12,000× *g*. The supernatant (1 mL) was mixed with 5 mL of 0.4 mol/L Na_2_CO_3_ and 1 mL of Folin–phenol reagent and incubated at 40 °C for 20 min. Then, the absorbance at 680 nm was measured using a spectrophotometer. One unit of protease activity was defined as the amount of enzyme required to produce 1 µg of tyrosine per minute.

### 2.3. Re-Screening of Isolates Suitable for the Preparation of B. papyrifera Feed

After selected isolates and type strains were individually cultivated under their corresponding conditions, 0.6% (*v*/*v*) inocula were inoculated into the *B. papyrifera* powder medium containing 0.1 g of brown sugar, 50 g of *B. papyrifera* powder, and 50 mL of water. They were then thoroughly mixed, transferred into plastic bags equipped with a one-way valve, and incubated for 6 d. The fermented feed obtained was heated at 105 °C for 15 min and incubated at 60 °C until dried. The *B. papyrifera* powder medium without any inocula was used as a control. The acid-soluble protein content in dried samples was determined based on Chinese agricultural standard NY/T 3801-2020 [22] by Pony Testing International Group (Qingdao, China), while free ammonia nitrogen content was measured according to Chinese national standard GB/T 12143-2008 [23]. Briefly, ammonia nitrogen was determined using the formaldehyde titration method under neutral conditions.

### 2.4. Molecular Identification of Isolated Strains

Molecular identification was performed for the taxonomic characterization of isolated strains [24]. Total DNA of isolated strains was extracted using bacterial, yeast, and fungal genomic DNA extraction kits (TransGen Biotech Co., Ltd.; Beijing, China) according to the manufacturer’s instructions. With the genomic DNA as a template, 16S rDNA sequences of bacterial strains were amplified by PCR using universal primers (27F (5′-3′): AGAGTTTGATCCTGGCTCAG and 1492R (5′-3′): TACGGCTACCTTGTTACGACTT), while the ITS gene regions of yeast and fungal isolates were amplified using primers ITS1 (5′-3′: TCCGTAGGTGAACCTGCGG) and ITS4-R (5′-3′: TCCTCCGCTTATTGATATGC). The reaction mixture contained 0.2 μL of Taq Plus DNA Polymerase, 0.5 μL of genomic DNA, 2.5 μL of 10 × PCR buffer, 1.0 μL of dNTP-mix, 1.0 μL of each primer, and 18.8 μL of ddH_2_O. PCR was carried out with 30 cycles of 95 °C for 5 min, 94 °C for 30 s, 57 °C for 30 s, and 72 °C for 90 s, with a final extension step at 72 °C for 10 min. All PCR products were then sequenced using the Sanger method with M13 universal primers at Sangon Biotech Co., Ltd. The sequencing results obtained were analyzed via nucleotide BLAST on the NCBI website (https://blast.ncbi.nlm.nih.gov/Blast.cgi, accessed on 19 August 2025), using a 97% percent identity threshold and an e-value smaller than 0.01, and the sequences obtained were aligned and compared using ClustalW software (version 2.1) embedded in MEGA 11.0. Finally, phylogenetic trees were constructed in MEGA 11.0 using the neighbor-joining method [25]. The confidence values of the branches of the phylogenetic trees were determined through bootstrap analyses based on 1000 resamplings.

### 2.5. Single-Factor Experiments for Improving the Quality of Fermented B. papyrifera Feed

#### 2.5.1. Effect of Cultivation Temperature on the Acid-Soluble Protein Content

After cultivating *Paenibacillus* sp. AY and *R. mucilaginosa* FG in TYC medium and malt extract broth, respectively, to the late logarithmic growth phase, they were mixed at a ratio of viable cells of 1:1, and 0.6% (*v*/*v*) inocula were inoculated into the *B. papyrifera* powder medium as previously described. The initial coculture conditions were a 37 °C cultivation temperature, 5 d fermentation time, 50 g of *B. papyrifera* leaf powder, and 50 mL of water. This experimental setup involved incubation at five distinct temperatures (28, 31, 34, 37, and 40 °C), while all other conditions were the same as the initial conditions. After 5 days of fermentation, samples were taken to determine the acid-soluble protein content.

#### 2.5.2. Influence of Fermentation Time on the Acid-Soluble Protein Content

After 0.6% (*v*/*v*) inocula were inoculated into the *B. papyrifera* powder medium, they were cultured at 37 °C for 4, 5, 6, 7, and 8 days while keeping other conditions the same as described above. After 5 days of incubation, samples were withdrawn to determine the acid-soluble protein content.

#### 2.5.3. Influence of Loading Amount on Acid-Soluble Protein Content

*B. papyrifera* powder media composed of 40, 45, 50, 55, and 60 g *B. papyrifera* powder were individually used to prepare the feed while keeping all other conditions at a fixed level. After 5 days of cultivation, the acid-soluble protein content was assessed.

#### 2.5.4. Effect of Water Content on Acid-Soluble Protein Content

The volume of water was varied at 40, 45, 50, 55, and 60 mL to investigate its influence on the acid-soluble protein content. All other components were maintained at fixed levels.

### 2.6. Orthogonal Experimental Design of Fermentation Conditions

Based on the results of single-factor experiments, three process parameters were determined to be influencing factors: fermentation temperature (A), fermentation time (B), and water content (C). Three values were set for each influencing factor; the values of each level for each factor are shown in Table 1. Considering the number of experiments and the reliability of the experimental results, a 3-factor, 3-level experimental plan was adopted in this study, using an L9 (3^3^) orthogonal table. A linear model was used for the statistical analysis of each variable.

### 2.7. Statistical Analyses

All experiments were performed in triplicate, with results expressed as the mean ± standard deviation (SD). The figures were drawn in Origin (version 2022; OriginLab Corporation, Northampton, MA, USA), and data were statistically analyzed using SPSS software (version 2019b; SPSS Inc., Chicago, IL, USA). Results were considered significantly different when *p*-values were less than 0.05. The least significant difference (LSD) test was used to examine the differences between the parameters.

### 2.8. Nucleotide Sequence Accession Numbers

The ribosomal DNA sequence of *Paenibacillus* sp. AY and the ITS gene sequence of *R. mucilaginosa* FG have been deposited in the GenBank database under accession numbers PX112460 and PX112467, respectively. The accession numbers for the other eight bacterial isolates and four yeast/fungal strains are PX496875-PX496882 and PX496887-PX496890, respectively.

## 3. Results

### 3.1. Primary and Secondary Screening of Protease-Harboring Microorganisms

To isolate protease-active bacteria, all samples were cultured in the enrichment medium and then spread onto the screening medium containing skim milk powder; after one day of cultivation at 37 °C, the sizes of the colonies and transparent zones formed by each bacterial strain were measured. As shown in Table 2, isolates AH, AK, AL, AM, AS, AT, AV, AW, and AY exhibited ratios of transparent zone diameter to colony diameter (D/d) greater than 6.0, indicating their proficiency in protein degradation. Conversely, isolates that failed to display transparent zones were deemed incapable of hydrolyzing proteins and consequently excluded from further utilization. The initially screened isolates with transparent zones were cultivated in nutrient broth medium. Subsequent determination of protease activity demonstrated that isolates AL (62.82 U/mL), AM (65.21 U/mL), AS (67.01 U/mL), and AW (62.03 U/mL) had enhanced activities compared to the other isolates (Table 2).

**Figure 1:**
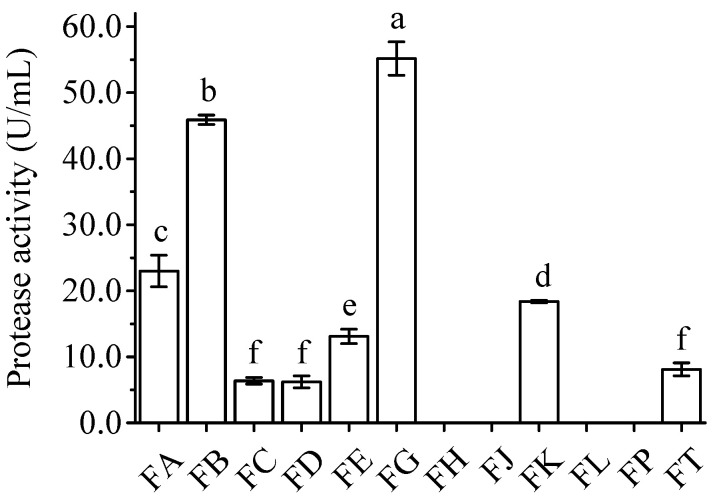
Protease activities of various yeast and fungal strains. Different superscript lowercase letters represent significant differences (*p* < 0.05).

To screen protease-harboring yeasts and fungi, all samples were cultivated in enrichment medium and subsequently spread onto Rose Bengal medium. After cultivation at 30 °C for 3 days, single yeast colonies or mycelia were individually inoculated into freshly prepared malt extract broth. After incubation, samples were taken to investigate their protease activities. As shown in Figure 1, strains FB and FG demonstrated proteolytic activities of 45.90 and 55.16 U/mL, respectively, significantly higher (*p* < 0.05) than those of the other isolates, indicating that these strains possess potentially robust protease productivity with efficient protease secretion activity. Conversely, no protease activity was detected in isolates FH, FJ, FL, and FP.

### 3.2. Re-Screening of Appropriate Strains for the Efficient Preparation of B. papyrifera Feed

To further investigate the effects of bacterial strains on the quality of fermented *B. papyrifera* feed, all selected isolates and the type strain *B. subtilis* AS 1.398 were individually inoculated into *B. papyrifera* powder medium. The acid-soluble protein and ammonia nitrogen contents were determined after 6 days of fermentation. Compared with the control, all tested strains significantly enhanced the acid-soluble protein and ammonia nitrogen contents (*p* < 0.05, Table 3). Moreover, all strains led to higher acid-soluble protein content in the fermented feed than the type strain *B. subtilis* AS 1.398, while the effects of isolates AK, AW, and AY on the ammonia nitrogen content were similar to those of the type strain.

Similarly, the effects of yeast and fungal strains on the quality of fermented *B. papyrifera* feed were also studied. As shown in Table 4, all isolates significantly (*p* < 0.05) improved the acid-soluble protein and ammonia nitrogen contents in the fermented feed compared to the control. Among isolates, FG outperformed the type strains *S. cerevisiae* S288C and *A. oryzae* RIB40 in terms of acid-soluble protein and ammonia nitrogen contents of the fermented *B. papyrifera* feed.

### 3.3. Molecular Identification

**Figure 2:**
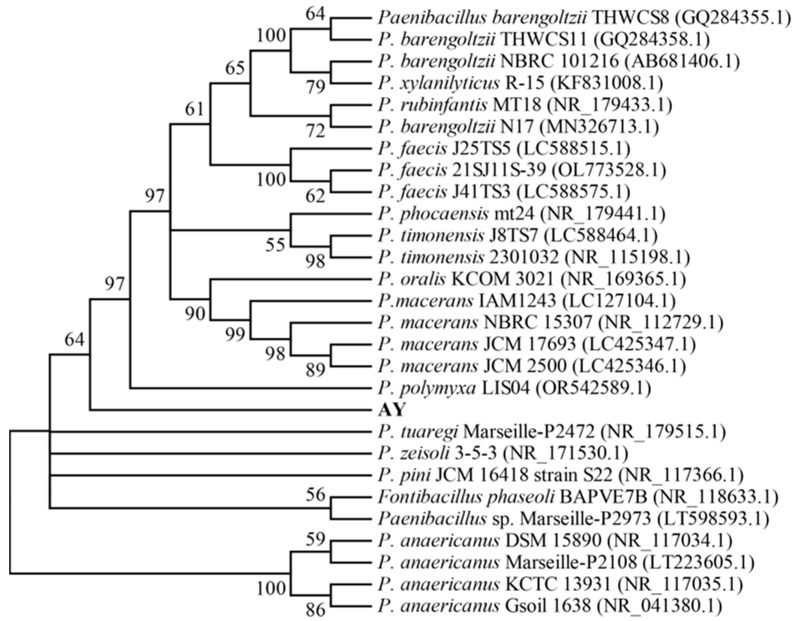
Phylogenetic analysis of bacterial strain AY. This is a neighbor-joining tree constructed using MEGA 11 software based on 16S rRNA sequences. Bootstrap resampling was conducted with 1000 pseudoreplicates to assess support for each individual branch; values lower than 50% are not shown. The total length of the sequences is 1483 bp, containing 297 phylogenetically informative characters.

The ribosomal DNA sequences of bacterial isolates were compared with those deposited in the NCBI database. AH, AL, and AV had high homology to *Stenotrophomonas maltophilia* (GenBank accession NO. KT748642.1), and strains AM, AS, and AW shared high similarity with *Aeromonas hydrophila* (accession NO. OL774807.1), while AT and AK displayed high homology to *B. anthracis* (accession NO. OQ293911.1). Therefore, all eight bacterial strains may be opportunistic pathogens. In addition, isolate AY shared high homology (96.51%) with *Paenibacillus tuaregi* Marseille-P2472 (accession NO. NR_179515.1), as depicted by the results of phylogenetic analysis in Figure 2. Based on all of these findings, strain AY is inferred to belong to the genus *Paenibacillus* and has been designated as *Paenibacillus* sp. AY, with an assigned accession number of PX112460 in the GenBank database.

**Figure 3:**
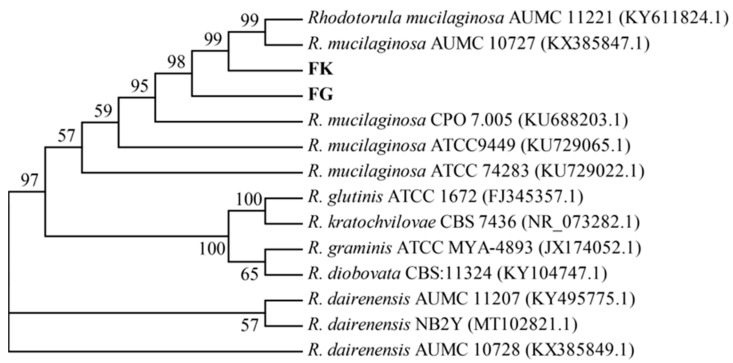
Phylogenetic analysis of fungal strains FG and FK. This is a neighbor-joining tree constructed using MEGA 11 software based on the ITS gene sequences. Bootstrap resampling was conducted with 1000 pseudoreplicates to assess support for each individual branch; values lower than 50% are not shown. The total length of the sequences is 579 bp, containing 124 phylogenetically informative characters.

The sequencing results of yeast and fungal isolates were also analyzed by comparing them to the NCBI BLAST database. Isolates FA, FB, and FE were found to be highly homologous to *Plectosphaerella cucumerina* W1-6 (accession NO. MK773577.1), *P. cucumerina* W1-6 (accession NO. MK773577.1), and *Cryptococcus laurentii* R134 (accession NO. HG532080.1), respectively. Isolates FG and FK shared high similarity with *Rhodotorula mucilaginosa* AUMC 10727 (accession NO. KX385847.1), which was further confirmed by phylogenetic analysis (Figure 3). Therefore, isolate FG was designated as *R. mucilaginosa* FG, and its ITS gene sequence has been deposited in the GenBank database under the accession number of PX112467.

Based on the results of primary and secondary screening, as well as re-screening, the isolates *Paenibacillus* sp. AY and *R. mucilaginosa* FG not only exhibited higher proteolytic activities but also increased the fermentation quality of *B. papyrifera* feed. Therefore, they were selected for subsequent studies.

### 3.4. Single-Factor Experiments Using Paenibacillus sp. AY and R. mucilaginosa FG for Optimization of B. papyrifera Feed Preparation Process

**Figure 4:**
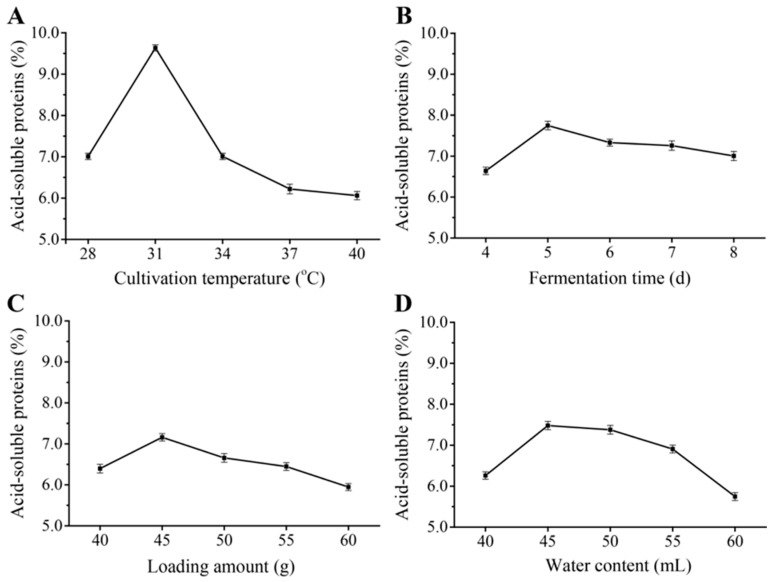
Effects of cultivation temperature (**A**), fermentation time (**B**), loading amount (**C**), and water content (**D**) on acid-soluble protein content in the fermented feed. The *B. papyrifera* feed was prepared by coculturing *Paenibacillus* sp. AY and *R. mucilaginosa* FG under initial conditions of 37 °C cultivation temperature, 5 d fermentation time, 50 g of *B. papyrifera* leaf powder, and 50 mL of water. (**A**) The two strains were cocultured at 28, 31, 34, 37, and 40 °C while keeping all other conditions at fixed levels and (**B**) cultured for 4, 5, 6, 7, and 8 days. (**C**) *B. papyrifera* powder media composed of 40, 45, 50, 55, and 60 g of *B. papyrifera* powder were each used to prepare the feed, while keeping all other conditions at fixed levels. (**D**) The volume of water was varied at 40, 45, 50, 55, and 60 mL, while all other components were maintained at fixed levels.

The “change one factor at a time” method was employed to investigate the impacts of culture temperature, fermentation time, loading amount, and water content on the quality of fermented *B. papyrifera* feed. Figure 4A illustrates the effects of culture temperature on the quality of *B. papyrifera* feed. The acid-soluble protein content reached a maximum of 9.63% after 5 days of incubation at 31 °C, but decreased sharply when the fermentation temperature was below or above this temperature. Therefore, 31 °C was regarded as the optimal culture temperature for *B. papyrifera* feed preparation using the present isolates.

The impact of fermentation time on the quality of fermented *B. papyrifera* feed was similarly investigated at 37 °C. As shown in Figure 4B, the acid-soluble protein content reached a maximum of 7.75% after 5 days of incubation but decreased when the fermentation time was shorter or longer than this time. Accordingly, a 5-day incubation time was considered optimal for the preparation of *B. papyrifera* feed using these isolates.

The effects of the loading amount of *B. papyrifera* leaf powder on the quality of *B. papyrifera* feed were then studied. The acid-soluble protein content in the fermented feed exhibited a similar trend of first increasing and then decreasing, with a maximum of 7.16% when 45 g of *B. papyrifera* leaf powder was added (Figure 4C). Consequently, this amount emerged as the most effective experimental condition for optimal fermented *B. papyrifera* feed preparation.

Finally, the influence of water content on the quality of *B. papyrifera* feed was investigated. As shown in Figure 4D, the optimal water content was 45 mL, as the acid-soluble protein content decreased when water content was lower or higher than this value. Thus, 45 mL of water was considered optimal for enhancing the acid-soluble protein content in fermented *B. papyrifera* feed.

### 3.5. Orthogonal Experimental Design for Optimizing B. papyrifera Feed Preparation Process

From the results of single-factor experiments, culture temperature, fermentation time, and water content were identified as influencing factors. Thus, a three-factor, three-level orthogonal experimental design (Table 1) was adopted to investigate the interactions between these dominant factors and optimize the fermentation process. The range analysis demonstrated that the factors’ order of influence on the acid-soluble protein content is A > B > C, corresponding to culture temperature > fermentation time > water content (Table 5). Based on mean value (*K*) analysis, the optimal combination was determined to be A_2_B_2_C_3_, representing the following conditions: 31 °C cultivation temperature, 5 d fermentation, and 50 mL of water. Under these optimal conditions, triplicate experiments yielded an acid-soluble protein content of 7.63 ± 0.02%, which was 27.2% higher than the initial result.

## 4. Discussion

Over the past few decades, *B. papyrifera* has received significant research attention due to its wide distribution, high yield, strong environmental adaptability, comprehensive nutrition, and high protein content. To make full use of this plant in the feed industry, especially its crude proteins, microbial fermentation represents a promising technique [26]. To date, most research concerning the preparation of *B. papyrifera* feed has been centered on commercial microorganisms, which may not be suitable for the fermentation of *B. papyrifera* and may prevent the efficient utilization of its crude proteins. Thus, isolating the appropriate microbial strain and establishing an optimal fermentation process has become one of the most important issues for the efficient preparation of *B. papyrifera* feed [27]. In the present study, using the root zone soil of *B. papyrifera* as the starting material, nine bacterial and eight yeast and fungal strains with relatively high protease activities were isolated and screened using qualitative and quantitative methods (Table 2 and Figure 1). Further analysis showed that bacterial isolate AY and fungal isolate FG could significantly (*p* < 0.05) increase the acid-soluble protein and ammonia nitrogen contents in fermented *B. papyrifera* feed compared to the control, with levels higher than or similar to those obtained with type strains (Table 3 and Table 4). Finally, these two strains were identified and designated as *Paenibacillus* sp. AY and *R. mucilaginosa* FG (Figure 2 and Figure 3), respectively. Genome sequencing and annotation revealed that these two strains do not encode toxic genes. To our knowledge, this is the first report on screening appropriate microbial strains from the root zone soil of *B. papyrifera* for the efficient utilization of its crude proteins.

Previous evidence has shown that *Paenibacillus* sp. strains produce various antimicrobials, exopolysaccharides, and hydrolytic enzymes, which offer protection against many pathogens of livestock and poultry [28]. Many of these strains have already been adopted as feed additives to enhance the growth performance of animals, including *P. polymyxa* AM20 [29], *P. pabuli* E1 [30], and *P. konkukensis* SK 3146 [31]. Furthermore, Yue et al. demonstrated that *P. polysaccharolyticus* XY5 promotes the degradation of fiber components in a co-fermentation system of mulberry leaves and distillers’ grains [32]. *R. mucilaginosa* strains can also produce cartenoids, beta-carotene, astaxanthin, single-cell proteins, lipids, enzymes, and other functional bioproducts from low-cost agricultural waste materials [33,34]. In addition, dietary supplementation with *R. mucilaginosa* strains could enhance the growth performance, antioxidant capacity, and immune function of animals, as well as modulate the composition of their gut microbiota [35]. The extract of *R. mucilaginosa* sp. GUMS16 [36] and *R. mucilaginosa* P22 [37] exhibited anti-tumor, and antibacterial and wound healing activities, respectively. Therefore, *Paenibacillus* sp. and *R. mucilaginosa* strains exhibit huge developmental and application potential and can be used for the fermentation of *B. papyrifera* feed.

Fermentation parameters significantly affect the quality of fermented feed, and various methods can be adopted to optimize the process. In a previous study, Zhang et al. used an orthogonal experimental design to optimize fermentation conditions to increase the quality of fermented *B. papyrifera* feed, whereas Lv et al. simultaneously employed single-factor experiments and the response surface method to optimize the fermentation conditions of *B. papyrifera* feed (Table 6). In this study, the coculture conditions of the isolates *Paenibacillus* sp. AY and *R. mucilaginosa* FG were optimized using single-factor experiments and an orthogonal design by varying the culture temperature, fermentation time, loading amount, and water content. The highest acid-soluble protein content was obtained at a cultivation temperature of 31 °C, fermentation time of 5 d, water content of 50 mL, and loading amount of 45 g (Figure 4 and Table 5). Under these conditions, the acid-soluble protein content reached 7.63%, which is higher than the result obtained by Lv et al. (6.84%) but much lower than that reported by Zhang et al. (12.56%). This may be attributable to different initial contents of acid-soluble proteins in the *B. papyrifera* leaf powder in these studies. Future research should focus on adopting the surface response method, genetic algorithm, and neural network model to further optimize the fermentation process of *B. papyrifera* feed. In addition, proteases involved in the degradation of crude proteins in *B. papyrifera* leaf powder should be identified by using omics technologies, such as transcriptomics and proteomics.

## 5. Conclusions

This study has demonstrated that the two selected isolates, designated *Paenibacillus* sp. AY and *R. mucilaginosa* FG, can effectively degrade crude proteins in *B. papyrifera* leaf powder and increase its fermentation quality. Single-factor optimization identified a 31 °C cultivation temperature, 5 d fermentation time, 45 g of *B. papyrifera* leaf powder, and 50 mL of water as favorable culture conditions for maximizing acid-soluble protein content with these isolates. Furthermore, the quality of fermented *B. papyrifera* feed was substantially improved by applying an orthogonal experimental design. Under the optimal conditions, the acid-soluble protein content reached a maximum of 7.63 ± 0.02%. Hence, this study not only identifies promising strains for *B. papyrifera* feed preparation, greatly improving the utilization efficiency of crude proteins, but also provides a valuable reference for screening and cultivating similar strains in future research endeavors.

## Figures and Tables

**Table 1:** Factors and levels of the orthogonal experimental design.

Levels	Factors		
	Fermentation Temperature (A)	Fermentation Time (B)	Water Content (C)
1	28	4	40
2	31	5	45
3	34	6	50

**Table 2:** Screening of protease-producing bacteria using the plate assay and enzymatic activity assay methods.

Isolates	Diameter of Transparent Zone (D, mm)	Diameter of Colony (d, mm)	Ratio (D/d)	Activity of Proteases (U/mL)
AH	7.5	1.1	6.82	20.05 ± 2.84 ^d^
AK	12.2	1.5	8.13	44.70 ± 3.52 ^b^
AL	10.3	1.0	10.30	62.82 ± 3.24 ^a^
AM	11.6	1.2	9.67	65.21 ± 1.27 ^a^
AS	12.7	2.0	6.35	67.01 ± 2.96 ^a^
AT	11.5	1.5	7.67	30.03 ± 1.01 ^c^
AV	11.6	1.6	7.25	48.49 ± 2.11 ^b^
AW	13.4	1.6	8.38	62.03 ± 0.99 ^a^
AY	10.1	1.5	6.73	21.95 ± 0.14 ^d^

Data with different superscripts in the same column are significantly different from each other (*p* < 0.05), while those with the same superscripts are not significantly different from each other (*p* > 0.05).

**Table 3:** Effects of different bacterial strains on the quality of fermented *B. papyrifera* feed.

Isolates	Acid-Soluble Protein Content (%)	Ammonia Nitrogen Content (mg/100 g)
Control	5.96 ± 0.09 ^i^	95.2 ± 0.3 ^i^
AH	8.41 ± 0.01 ^c^	115.6 ± 0.4 ^e^
AK	7.61 ± 0.13 ^d^	108.5 ± 0.7 ^f^
AL	7.00 ± 0.06 ^g^	129.5 ± 1.3 ^b^
AM	7.24 ± 0.13 ^g^	115.5 ± 1.1 ^e^
AS	8.78 ± 0.10 ^b^	125.8 ± 0.9 ^c^
AT	7.83 ± 0.13 ^de^	119.0 ± 1.2 ^d^
AV	7.23 ± 0.06 ^g^	133.0 ± 0.7 ^a^
AW	9.06 ± 0.06 ^a^	105.4 ± 1.4 ^fh^
AY	7.55 ± 0.06 ^df^	106.8 ± 1.1 ^f^
*B. subtilis* AS 1.398	6.42 ± 0.07 ^h^	109.4 ± 1.5 ^fg^

The *B. papyrifera* powder medium without any inocula was used as a control. Data with different superscripts in the same column are significantly different from each other (*p* < 0.05), while those with the same superscripts are not significantly different from each other (*p* > 0.05).

**Table 4:** Effects of different yeast and fungal strains on the quality of fermented *B. papyrifera* feed.

Isolates	Acid-Soluble Protein Content (%)	Ammonia Nitrogen Content (mg/100 g)
Control	4.76 ± 0.01 ^e^	166.6 ± 1.3 ^f^
FA	5.89 ± 0.04 ^d^	179.3 ± 1.5 ^e^
FB	6.26 ± 0.21 ^c^	181.5 ± 1.7 ^d^
FE	6.61 ± 0.09 ^abc^	197.2 ± 0.9 ^c^
FG	6.82 ± 0.04 ^a^	207.4 ± 1.1 ^ab^
FK	6.48 ± 0.07 ^bc^	210.8 ± 0.7 ^a^
*S. cerevisiae* S288C	6.79 ± 0.03 ^a^	204.0 ± 1.2 ^b^
*A. oryzae* RIB40	6.39 ± 0.01 ^c^	200.6 ± 1.1 ^bc^

The *B. papyrifera* powder medium without any inocula was used as a control. Data with different superscripts in the same column are significantly different from each other (*p* < 0.05), while those with the same superscripts are not significantly different from each other (*p* > 0.05).

**Table 5:** Results of orthogonal experimental design.

Number	A (°C)	B (d)	C (mL)	Acid-Soluble Protein Content (%)
1	28	4	40	6.99 ± 0.03 ^a^
2	28	5	45	7.08 ± 0.02 ^ab^
3	28	6	50	7.13 ± 0.03 ^c^
4	31	4	45	7.54 ± 0.03 ^e^
5	31	5	50	7.63 ± 0.02 ^a^
6	31	6	40	7.58 ± 0.01 ^b^
7	34	4	50	6.84 ± 0.02 ^d^
8	34	5	40	6.88 ± 0.03 ^e^
9	34	6	45	6.79 ± 0.03 ^f^
*k*1	7.07	7.12	7.15	
*k*2	7.58	7.2	7.14	
*k*3	6.84	7.17	7.2	
*R*	0.71	0.06	0.05	

Data with different superscripts in the same column are significantly different from each other (*p* < 0.05), while those with the same superscripts are not significantly different from each other (*p* > 0.05).

**Table 6:** Comparisons between this study and other research studies.

	Optimization Method	Acid-Soluble Protein Content (%)	Reference
This study	Single-factor experiments and orthogonal design	7.63	
Lv et al.	Single-factor experiments and response surface method	6.84	[27]
Zhang et al.	Orthogonal design	12.56	[38]

## Data Availability

The data presented in this study are openly available in NCBI database (https://www.ncbi.nlm.nih.gov/) under accession numbers PX112460, PX112467, PX496875-PX496882 and PX496887-PX496890.
